# RETIREMENT TIMING AND POST-RETIREMENT HEALTH: QUASI-EXPERIMENTAL EVIDENCE FROM DENMARK

**DOI:** 10.1093/geroni/igz038.3294

**Published:** 2019-11-08

**Authors:** Jeevitha Y Qvist

**Affiliations:** Department of Political Science, Aalborg University, Aalborg, Denmark

## Abstract

A growing concern among policy makers in the European welfare states is that the proportion of the population in the working age has decreased over the last decades. In response to these demographic trends, many European countries have introduced reforms that roll back welfare policies that enables early retirement in order to sustain the current standards of living. However, scholars have voiced the concern that reforms which prevent early retirement could cause a rise in health inequality in old age because some people are not able to extend their working life. There are two contradictory views on post-retirement health. Retirement can either be seen as a kind of identity crisis, leading to less motivation to maintain health or retirement can be seen as a health preserving transition, enabling individuals to relieve stress and be more aware of their health. So far, empirical evidence on the effect of retirement timing on post-retirement health is inconclusive about the causal nature of this relationship. To estimate the causal effect of retirement timing on post-retirement health, this paper uses month of birth variation in incentives to postpone early retirement in the cohort born in 1939 that was created by a reform of the Danish retirement legislation, which the government introduced in 1999. The results suggest that people who retire at the age of 60 have more adverse health outcomes in old age than people who retire later, but this difference does not appear to be caused by differences in retirement timing.

